# The lncRNA B3GALT5-AS1 Functions as an HCC Suppressor by Regulating the miR-934/UFM1 Axis

**DOI:** 10.1155/2021/1776432

**Published:** 2021-10-20

**Authors:** Erlin Chen, Bin Zhou, Saiyan Bian, Wenkai Ni, Zhong Chen

**Affiliations:** ^1^Department of General Surgery, The First Affiliated Hospital of Soochow University, Soochow University, Suzhou, Jiangsu 215123, China; ^2^Department of General Surgery, Affiliated Hospital of Nantong University, Nantong, Jiangsu 226001, China; ^3^Departments of General Surgery, Suzhou Wuzhong People's Hospital, Suzhou, Jiangsu 215100, China; ^4^Department of Gastroenterology, Affiliated Hospital of Nantong University, Nantong, Jiangsu 226001, China; ^5^Medical College, Nantong University, Nantong, Jiangsu 226001, China; ^6^Endoscopy Center and Endoscopy Research Institute, Zhongshan Hospital, Fudan University, Shanghai 200032, China

## Abstract

Accumulating evidence has demonstrated that long noncoding RNA (lncRNA) is importantly related to the occurrence and development of cancer. According to reports, the expression of B3GALT5-AS1 in hepatocellular carcinoma (HCC) is downregulated; however, the role of B3GALT5-AS1 in HCC is not yet clear. In this study, our purpose is to explore the biological function of B3GALT5-AS1 in HCC and its coupling mechanism with miR-934 and ubiquitin-fold modifier 1 (UFM1). We found that the B3GALT5-AS1 expression level was of significant reduction in both HCC tissues and cell lines; B3GALT5-AS1 overexpression (ov) may inhibit the malignant features of HCC. In addition, we demonstrated that miR-934 mimics could reverse the effect of B3GALT5-AS1 ov, which proved miR-934 was the downstream regulator of B3GALT5-AS1. Furthermore, si-UFM1 could reverse the effect of miR-934 inhibitor, which revealed the connection between them. Moreover, we found that B3GALT5-AS1 could keep down the PI3K/AKT pathway through UFM1. Our results demonstrated that B3GALT5-AS1 was an excellent HCC suppressant by regulating miR-934 and UFM1 to achieve negative regulation of HCC cell proliferation, invasion, and metastasis, indicating that B3GALT5-AS1 is a promising potential therapeutic target for HCC treatment.

## 1. Introduction

In recent years, the incidence and mortality of tumors have been increasing, and hepatocellular carcinoma (HCC) as a primary liver cancer with high mortality has attracted widespread attention [1]. Its incidence ranks 6th among systemic malignancies, and its mortality ranks 3rd. With the change of people's dietary habits and environmental pollution intensified, the incidence of HCC worldwide is rising rapidly, and the number of patients who die from HCC each year is as high as 800,000 [2, 3]. At present, the etiology and exact molecular mechanism of HCC are not fully understood [4, 5]. It is of great significance to find effective therapeutic targets and to explore the molecular mechanisms of HCC.

Long noncoding RNA (lncRNA) is a type of RNA molecules with a transcript length of more than 200 nt. It is generally believed that they do not encode proteins but participate in the regulation of protein-coding genes at the RNA level [6, 7]. Research results in recent years have shown that lncRNA can participate in the biological processes of cell proliferation, migration, and apoptosis of many tumors including liver cancer through epigenetic regulation, transcription regulation, and post-transcriptional regulation [8–10]. LncRNA B3GALT5-AS1 is located on the 39597147–39612822 of chromosome 21q22.2, containing 4 exon regions; it is downregulated in multiple malignancies, such as colon cancer [11–13] and gastric cancer [14]. LncRNA B3GALT5-AS1 was reported that can repress cell growth and induce cell apoptosis. The downregulation of B3GALT5-AS1 promotes the tumorigenesis and cell invasion. These studies indicate that lncRNA B3GALT5-AS1 can act as a potential biomarker in cancer.

MicroRNA (miRNA) is a class of endogenous noncoding RNA found in eukaryotes that have the function of regulating post-transcriptional gene expression [15]. It has been reported that miRNA can specifically recognize the 3′-UTR end of target mRNA and form a single-stranded noncoding RNA with a length of 18–22 nucleotides. Then, the downstream target mRNA or the upstream gene is regulated and the translation process is blocked, which plays an important role in the behavior of malignant tumors [16]. More and more evidence has shown that noncoding RNAs such as lncRNA and miRNA are gradually emerging in the field of disease diagnosis and treatment and playing an important role in tumor regulation. The successful exploration of the interaction mechanism between the two may provide new ideas for the discovery of new tumor targets [17]. Ubiquitin-fold modifier 1 (UFM1), a ubiquitin-like protein, has recently been related to tumor progression also [18, 19]. However, the interaction between UFM1, miR-934, and lncRNA is not clear.

Here, we screened a newfangled lncRNA, B3GALT5-AS1, with lower expression functions as a HCC suppressor by regulating the miR-934 and UFM1 axis. The high expression of B3GALT5-AS1 in HCC cells is related to the good survival of HCC patients; overexpression of B3GALT5-AS1 could inhibit the malignant features of HCC. In addition, we demonstrated that B3GALT5-AS1 ov was negatively correlated with miR-934, miR-934 was negatively relevant to UFM1, and B3GALT5-AS1 ov was positively associated with UFM1. Moreover, we found that B3GALT5-AS1 could keep down the PI3K/AKT pathway through UFM1. These results indicate that B3GALT5-AS1 can be considered a potential therapeutic target for HCC.

## 2. Materials and Methods

### 2.1. Tissue Specimen

This research protocol was approved by the Affiliated Hospital of Nantong University's Ethics Committee, and a total of 56 pathological tissues of the patients were selected. The tumor tissues and matched adjacent tissues of all patients were freshly collected, and the adjacent tissues were more than 2 cm from the edge of the tumor. After the specimens were obtained, they were placed in a cryotube and immediately stored in liquid nitrogen.

### 2.2. Cell Cultures

Five HCC cell lines (Hep3B, Focus, YY-8103, HCCLM3, and Huh7) and normal liver cell line (L02) were obtained from the ATCC. Cells were cultured in DMEM containing 1% antibiotics (100 U/ml penicillin and 100 mg/ml streptomycin sulfates) and 10% fetal bovine serum (FBS) at 37°C in a humidified 5% CO_2_ atmosphere.

### 2.3. Cell Transfection

si-NC or si-B3GALT5-AS1 was cloned into PGK plasmids and transfected into Hep3B cells to knockdown B3GALT5-AS1, while the pcDNA 3.1 plasmids with full-length B3GALT5-AS1 (B3GALT5-AS1 ov) and related negative controls (NC) were transfected into HCCLM3 cells to upregulate B3GALT5-AS1 following Lipofectamine™ 3000, respectively. MiR-934 inhibitor/mimics and si-UFM1 were further transfected into HCCLM3 cells, and finally qRT-PCR was used to evaluate the transfection efficiency.

### 2.4. RNA Extraction and qRT-PCR

The TRIzol reagent method was used to isolate total RNA from cultured cells and tissues for subsequent qRT-PCR, and the purity and concentration were measured by using a UV spectrophotometer [20]. And, the reverse transcription kit instructions are referred for reverse transcription of RNA into cDNA. The expression of B3GALT5-AS1 and UFM1 was detected by the ABI 7900HT RealTime PCR system using SYBR Green assays, and GAPDH was used as the internal control. The expression of miR-934 was measured using TaqMan MicroRNA Assays, and U6 was treated as an internal control. The cycle threshold (Ct) value was measured for quantification, and the 2^−ΔΔCt^ method was used for normalization. Each experiment was performed in triplicate. The primer sequences have been provided in Table 1.

### 2.5. Fluorescence In Situ Hybridization (FISH) Assay

Hep3B cells were selected to determine the localization of B3GALT5-AS1; CY3-labeled probe for B3GALT5-AS1 was used. The nuclear stained by DAPI was blue under ultraviolet excitation, and the cy3 appears red. The detailed operations were followed under the official guideline.

### 2.6. CCK-8 Assay

After the logarithmic growth phase cells were prepared into a single cell suspension of 1 × 10^4^ cells/ml, 100 *μ*l per well of cells was inoculated into a 96-well plate and cultured at 37°C and 5% CO_2_ saturated humidity. Cell proliferation was detected by a Cell Counting Kit-8 (CCK-8) assay (Dojindo) following manufacturer's instructions. Finally, the cell viability was analyzed by detecting the absorbance (OD) of each group at 450 nm wavelength.

### 2.7. Cell Colony Formation Assay

After the cells in the logarithmic growth phase are made into a single cell suspension, 300 cells/well are seeded into a 6-well plate and cultured at 37°C and 5% CO_2_ saturated humidity. After 2 weeks, the cells were fixed with methanol and stained with 1% crystal violet for 15 min. Finally, the number of cell colonies was counted.

### 2.8. Cell Apoptosis

The apoptosis detection kit (KeyGEN) was used to detect cell apoptosis. The cells were incubated in a 6-well plate (1 × 10^5^ cells per well) to the logarithmic growth phase. Then, the cells were processed according to kit instructions and stored in 70% ethanol at 4°C overnight. After that, cell apoptosis was analyzed by flow cytometry (BD Biosciences, San Jose, USA).

### 2.9. Cell Migration and Invasion Assay

After the logarithmic growth phase cells were prepared into a single cell suspension of 1 × 10^4^ cells/ml, the chambers were divided into two groups, i.e., without Matrigel and with Matrigel, and cell migration and invasion were detected, respectively. The finally migrated cells were fixed with ethanol, and then the cells were counted after staining with 0.1% crystal violet. For the wound-healing assays, cells were seeded in a 24-well plate at density of 1 × 10^6^ and incubated for 24 h to adhere. Then, a 200 *μ*l sterile pipette tip was used to scrape the adherent cells to form fissures; they were washed three times with PBS, and the wound healing was observed under a microscope after incubating for 48 h. The finally migrated cells were fixed with ethanol and then imaged after staining with 0.1% crystal violet (Olympus, Japan).

### 2.10. RNA Immunoprecipitation

The Magna RIP RNA-Binding Protein Immunoprecipitation Kit was used for RIP assay. To detect the miRNA binding to lncRNA, Hep3B cells were lysed in RIP lysis buffer and incubated with a biotin-coupled probe of B3GALT5-AS1 or oligo probe that was prebound on magnetic beads. The purified miRNA was then subjected to qRT-PCR. To detect the lncRNA binding to miRNA, the biotin-coupled probe of miRNA-934 or Biotin-NC was processed through the same protocol.

### 2.11. Vector Construction and Luciferase Reporter Assay

The binding sites of B3GALT5-AS1 and UFM1 with miR-934 predicted by the bioinformatics tool were amplified by PCR, respectively. The amplified products were inserted into pGL3 plasmid to construct wild-type (wt) plasmids of B3GALT5-AS1 and UFM1, and the B3GALT5-AS1 and UFM1 mutant (mut) plasmids were constructed using a site-directed mutagenesis kit (Takara, Japan). For luciferase reporter assays, luciferase activity was measured through the Dual-Luciferase Reporter Assay system (Promega Corporation) after transfected the miR-934 mimics with the wild-type and mutant-type plasmids of the above configuration for 48 h.

### 2.12. Western Blot

The cells were lysed on ice, and the total cell protein was extracted by centrifugation. The protein concentration was detected by the BCA method, and the loading buffer was added to the protein sample and heated to denature. Proteins were analyzed by 10% SDS-PAGE gel and transferred to polyvinylidene fluoride (PVDF) membranes (Bio-Rad). Then, the membrane was incubated with primary antibodies against UFM1, p-PI3K, t-PI3K, p-AKT, t-AKT, Bcl-2, Bax, Vimentin, E-cadherin protein, or GAPDH (Abcam) overnight at 4°C, followed by incubating with conjugated secondary antibody for 2 h. Finally, the membrane was placed with the luminescent agent into the imager for imaging.

### 2.13. Mouse Xenograft Model

We used five-week-old BALB/C nude mice to establish animal models; the cells were cultured and collected after digestion and centrifugation at first and then inoculated subcutaneously with 200 *μ*l of each mouse at a density of 5 × 10^5^/100 *μ*l. In the process of tumor growth, the length (*L*) and width (W) were measured using a caliper, and the volume of the tumor was calculated using the following formula: V = (*L* × *W*^2^) × 0.5. 20 days after the injection, the animals were euthanized and tumor weight was measured. Animal experiments were approved by the Animal Ethics Committee of the Affiliated Hospital of Nantong University.

### 2.14. Statistical Analysis

SPSS 20.0 software and GraphPad 8.0 were used to statistically process the raw data obtained in the experiment, and the raw data obtained in the experiment were all expressed as mean ± standard deviation (SD). The independent sample *t*-test was used for the comparison between the two groups, and the one-way analysis of variance was used for the comparison between multiple groups. The Kaplan–Meier test and log-rank analysis were used for survival analysis. *P* < 0.05 means that the difference is statistically significant.

## 3. Results

### 3.1. B3GALT5-AS1 Is Downregulated in HCC Tissues and Cell Lines

**Figure 1:**
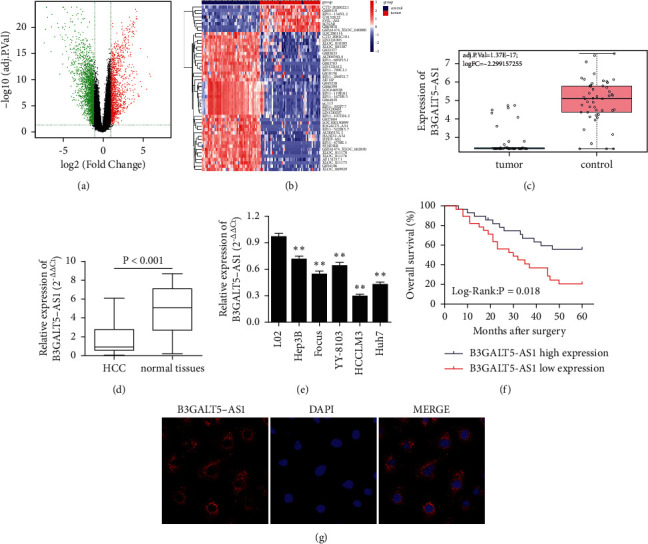
B3GALT5-AS1 was significantly downregulated in HCC and associated with higher survival time of HCC patients. (a) Screening of differential expression lncRNAs for HCC through the GEO database. (b) Top 50 different lncRNAs for liver cancer. (c) B3GALT5-AS1 expression in HCC tissues and adjacent normal tissues based on the GEO database. (d) B3GALT5-AS1 expression of 56 samples in HCC tissues and adjacent normal tissues. (e) B3GALT5-AS1 expression in the human normal liver cell line (L02) and five liver cancer cell lines (Hep3B, Focus, YY-8103, HCLM3, and Huh7). (f) Five-year survival period of 56 patients with low and high expression of B3GALT5-AS1. (g) FISH assay indicated that B3GALT5-AS1 was localized in the cytoplasm of Hep3B cell lines. ^*∗∗*^*P* < 0.01.

First, we screened differential expression lncRNAs for HCC through the GEO database (GSE138178, 49 HCC vs. 49 normal tissues) (Figure 1(a)), and the top 50 different lncRNAs for HCC are shown in Figure 1(b). Based on GEO data, we found that the expression of B3GALT5-AS1 in tumor tissues was lower than that of adjacent cancer tissues, indicating that high expression could suppress HCC progression (Figure 1(c)). To determine the expression of B3GALT5-AS1 in HCC tissues and adjacent normal tissues, we performed qRT-PCR in 56 HCC tissues and corresponding adjacent noncancerous tissues. As displayed in qRT-PCR assays, B3GALT5-AS1 expression was significantly downregulated in HCC tissues, with the average level over twofolds lower than that in normal tissues (Figure 1(d)). To examine B3GALT5-AS1 levels of HCC cell lines, qRT-PCR was performed in five human HCC cell lines (Hep3B, Focus, YY-8103, HCCLM3, and Huh7) and a control normal liver cell line (L02). The statistical analysis demonstrated that B3GALT5-AS1 expression was lower in HCC cells when compared with L02 cells and the expression in HCCLM3 and Hep3B was relatively lowest and highest, respectively (Figure 1(e)). Moreover, compared with the higher B3GALT5-AS1 expression group, the Kaplan–Meier curve analysis of 56 patients showed that the lower B3GALT5-AS1 expression group was associated with a worse survival time in HCC patients (Figure 1(f)). The result of FISH indicated that B3GALT5-AS1 was localized in the cytoplasm of Hep3B cell lines (Figure 1(g)). Furthermore, we studied the relationship between B3GALT5-AS1 expression and the clinicopathologic features of HCC patients. As shown in Table 2, B3GALT5-AS1 expression was significantly correlated to tumor size (*P*=0.022) and TNM stage (*P*=0.003). However, it was not related to age, gender, liver cirrhosis, HBsAg status, AFP, tumor multiplicity, and Edmondson grade.

### 3.2. B3GALT5-AS1 ov Inhibits HCCLM3 and Huh7 Cell Proliferation, Migration, and Invasion

**Figure 2:**
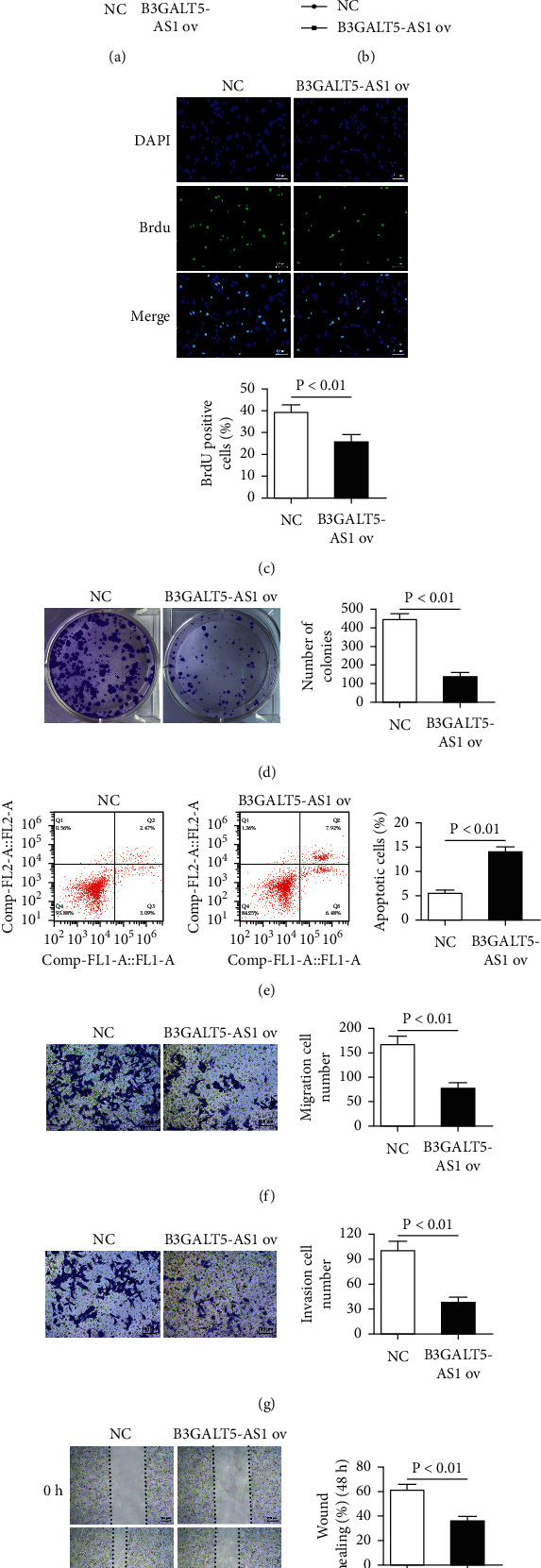
B3GALT5-AS1 overexpression inhibits HCCLM3 cell proliferation, migration, and invasion. (a) The expression level of B3GALT5-AS1 transfected with NC and B3GALT5-AS1 overexpression plasmid. Cell proliferation detected by the CCK-8 assay (b) and BrdU assay (c). (d) Cell colony formation assay of the NC group and B3GALT5-AS1 ov group. (e) Apoptosis assays of the NC group and B3GALT5-AS1 ov group. B3GALT5-AS1 ov decreased the migration (f) and the invasion (g) ability of HCCLM3 cells. (h) Wound-healing assays of the NC group and B3GALT5-AS1 ov group. ^*∗*^*P* < 0.05; ^*∗∗*^*P* < 0.01.

Given the downregulated B3GALT5-AS1 in both the HCC tissues and cell lines, we deduced that B3GALT5-AS1 may inhibit the growth of HCC. To clarify the biological function of B3GALT5-AS1 in HCC, we transfected the B3GALT5-AS1 overexpression plasmid into HCCLM3 cells and Huh7 cells, which were found to express the lower levels of B3GALT5-AS1 among the five HCC cell lines tested (Figure 1(e)). The transfection efficiency was confirmed by qRT-PCR, and the results demonstrated that B3GALT5-AS1 overexpression in HCCLM3 cells and Huh7 cells was successfully constructed. Obviously, the expression of B3GALT5-AS1 in the overexpression group was higher than that of NC group (*P* < 0.01, Figure 2(a)). CCK-8 assay showed that B3GALT5-AS1 ov dramatically suppressed cell proliferation in HCCLM3 cells and Huh7 cells compared with the NC groups (*P* < 0.05, Figure 2(b)). Furthermore, the BrdU cell proliferation assay was consistent with the results of CCK-8 assay, identifying the cell inhibitory function of B3GALT5-AS1 in HCCLM3 cells (*P* < 0.01, Figure 2(c)). The cell colony formation assay showed that cell colonies of B3GALT5-AS1 ov groups were smaller than those of NC groups (*P* < 0.01, Figure 2(d)). The experimental results above collectively illustrated that B3GALT5-AS1 was validated in suppressing the cell proliferation of HCC from CCK-8, BrdU, and colony formation assay. Moreover, we found that the overexpression of B3GALT5-AS1 markedly promoted the apoptosis and inhibited *in vitro* migration and invasion capabilities of HCCLM3 and Huh7 cells (*P* < 0.01, Figures 2(e)–2(g)). The wound-healing assay showed that the overexpression of B3GALT5-AS1 significantly inhibited the migration of HCCLM3 cells (*P* < 0.01, Figure 2(h)). Collectively, our research discovered that the overexpression of B3GALT5-AS1 can significantly inhibit the malignant characteristics of HCC.

### 3.3. B3GALT5-AS1 Knockdown Potentiated Hep3B and YY-8103 Cell Proliferation, Migration, and Invasion

**Figure 3:**
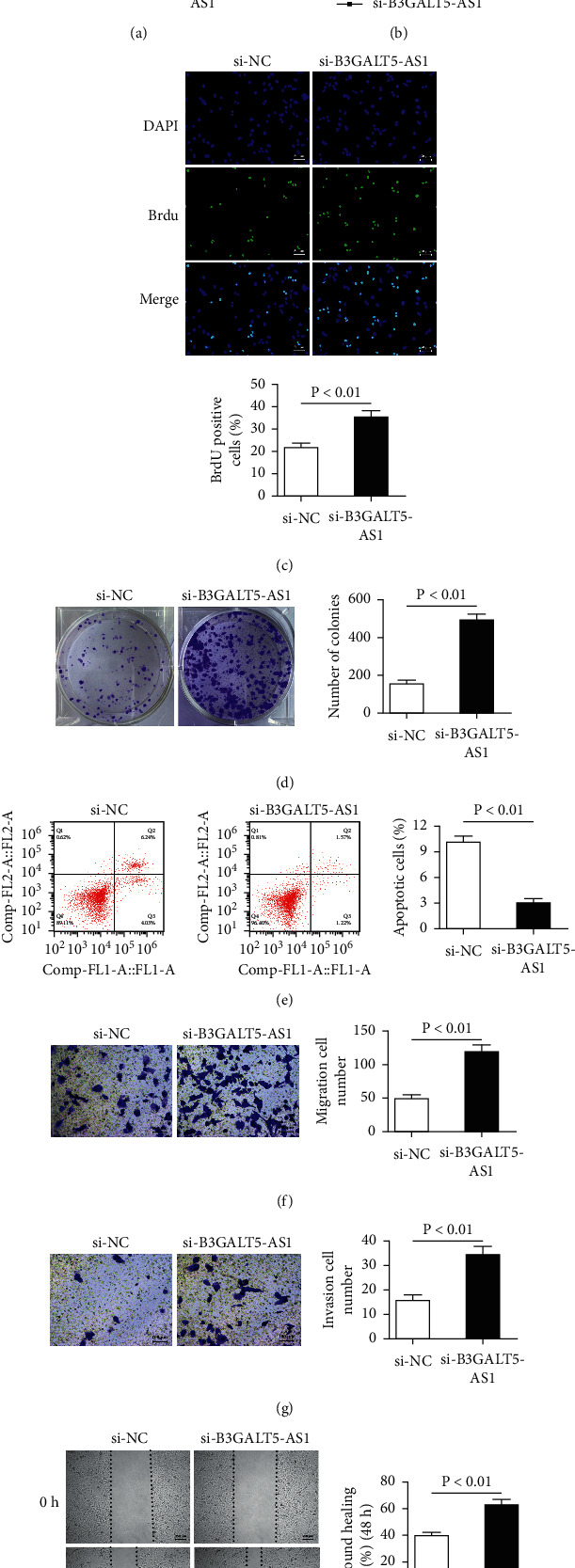
B3GALT5-AS1 knockdown promotes Hep3B cell progression. (a) B3GALT5-AS1 was downregulated in Hep3B cells transfected with si-B3GALT5-AS1 compared with cells transfected with si-NC. (b) CCK-8 assay was performed by transfection with si-NC and si-B3GALT5-AS1 into Hep3B cells. (c) Cell proliferation detected by the BrdU assay. (d) Cell colony formation assay of si-NC and si-B3GALT5-AS1-treated cells. (e) Apoptosis assays carried out in Hep3B cells. Migration (f) and invasion (g) assays in Hep3B cells transfected with si-NC and si-B3GALT5-AS1. (h) Representative images of wound-healing assays of cells transfected with si-NC and si-B3GALT5-AS1. ^*∗*^*P* < 0.05; ^*∗∗*^*P* < 0.01.

To further analyze the effects of B3GALT5-AS1 on proliferation, migration, and invasion, we chose Hep3B and YY-8103 cells to construct B3GALT5-AS1 knockdown cell lines by transfecting si-B3GALT5-AS1 (Figure 1(e)). The si-B3GALT5-AS1 was applied for B3GALT5-AS1 silencing in the following experiments based on a higher knockdown efficiency of B3GALT5-AS1 in Hep3B and YY-8103 cells. qRT-PCR was executed to judge the successful construction of B3GALT5-AS1 knockdown cell lines (*P* < 0.01; Figure 3(a)). The CCK-8 assay and BrdU assay observed that the knockdown of B3GALT5-AS1 promoted cell proliferation rate in Hep3B and YY-8103 cells (*P* < 0.05, *P* < 0.01; Figures 3(b) and 3(c)). Similarly, the effect of downregulation of B3GALT5-AS1 on cell colony formation and migration in the presence of si-B3GALT5-AS1 was tested. The results showed the cell colonies in Hep3B and YY-8103 cells were larger after B3GALT5-AS1 silence (*P* < 0.01; Figure 3(d)). Compared with the si-NC groups, the si-B3GALT5-AS1 groups showed observably downregulated apoptosis and induced *in vitro* migration and invasion of Hep3B and YY-8103 cells (*P* < 0.01, *P* < 0.01, *P* < 0.01; Figures 3(e)–3(g)). The migration of cells was measured by the wound-healing assay, and the results demonstrated that the knockdown of B3GALT5-AS1 significantly promoted the migration of Hep3B cells (*P* < 0.01; Figure 3(h)). Taken together, these results indicate that the knockdown of B3GALT5-AS1 may enhance the malignant features of HCC, including cell proliferation, migration, and invasion.

### 3.4. B3GALT5-AS1 ov and Knockdown Can Inhibit and Potentiate Xenograft Tumor Growth In Vivo, Respectively

**Figure 4:**
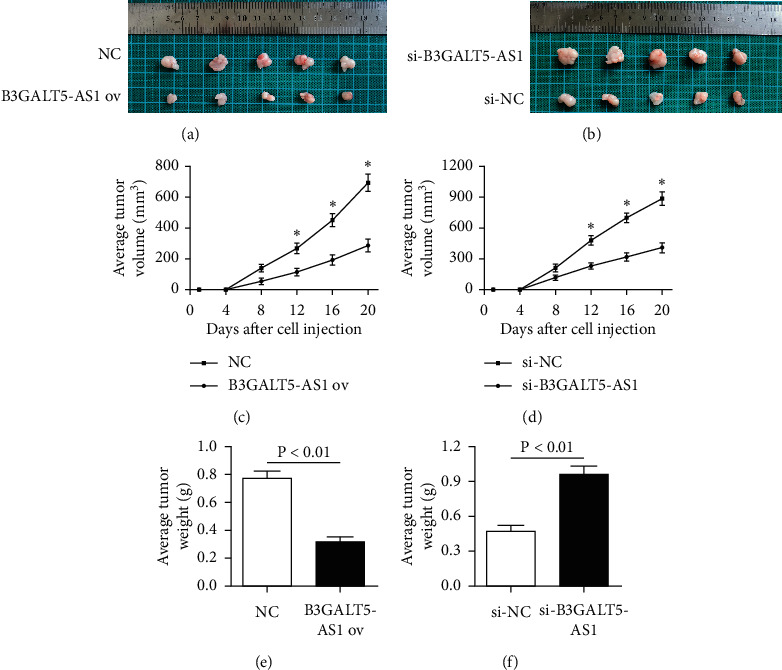
B3GALT5-AS1 overexpression and knockdown influences xenograft tumor growth in vivo. The mice of B3GALT5-AS1 overexpression groups (a) and si-B3GALT5-AS1 overexpression groups (b) were killed at 20 days after inoculation, and tumor tissues were excised and imaged. Average tumor volume of the overexpression group (c) and knockdown group (d) measured every four days. (e, f) Average tumor weight for each group. The overexpression of B3GALT5-AS1 significantly reduced tumor weight, and the tumor grew vigorously after B3GALT5-AS1 silencing compared with the NC groups. ^*∗*^*P* < 0.05; ^*∗∗*^*P* < 0.01.

To confirm whether B3GALT5-AS1 affects HCC *in vivo*, HCCLM3 and Hep3B cells were cultured and subcutaneously injected into mice to construct overexpression and knockdown models. In the present study, it was hypothesized that the downregulation of B3GALT5-AS1 may lead to cancer progression and the occurrence of a malignant phenotype. As shown in Figures 4(a) and 4(b), we peeled off the tumor from the mice and took a physical picture. The results showed that the overexpression of B3GALT5-AS1 significantly inhibited tumor growth; on the contrary, the tumor grew vigorously after B3GALT5-AS1 silencing compared with the NC group. To quantify the extent of tumor growth, we measured average tumor volume and average tumor weight of the overexpression and knockdown groups. Interestingly, B3GALT5-AS1 overexpression effectively inhibits tumor volume and weight compared with the NC treated group, and the opposite is true after knockdown (*P* < 0.05, *P* < 0.05, *P* < 0.01, *P* < 0.01, Figures 4(c)–4(f)). Taken together, B3GALT5-AS1 overexpression and knockdown could suppress and promote xenograft tumor growth *in vivo*, respectively.

### 3.5. B3GALT5-AS1 Targeted miR-934 Directly and Negatively Regulated miR-934

**Figure 5:**
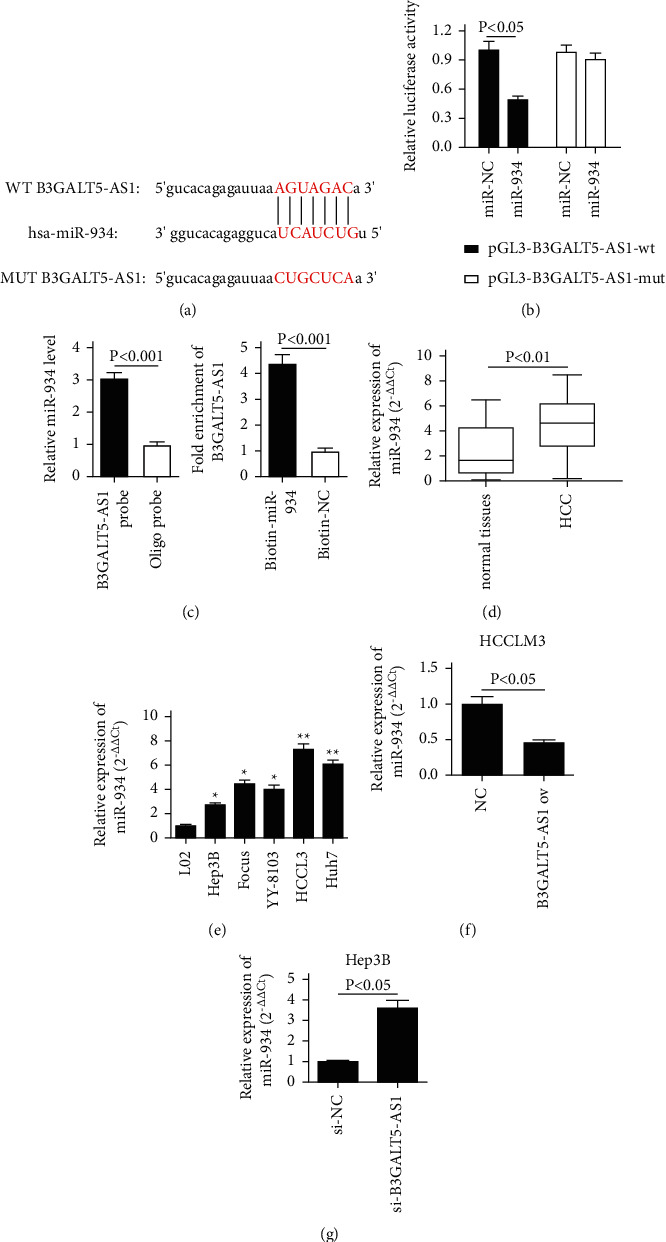
B3GALT5-AS1 targeted miR-934 directly and negatively regulated miR-934. (a) The wild-type (wt) and mutant-type (mut) complementary sequences of the B3GALT5-AS1 with miR-934. (b) Luciferase assays of HCC cells cotransfected with the pGL3-B3GALT5-AS1-wt or pGL3-B3GALT5-AS1-mut and miR-NC or miR-934. (c) RIP assay revealed that there was specific bind locations of B3GALT4-AS1 on miR-934. (d) The miR-934 expression in HCC tissues and adjacent normal tissues. (e) miR-934 expression in the L02 cell line and five liver cancer cell lines (Hep3B, Focus, YY-8103, HCLM3, and Huh7). (f, g) Expression of miR-934 of the NC group and B3GALT5-AS1 ov group in HCCLM3 cells and the si-NC group and si-B3GALT5-AS1 group in Hep3B cells. ^*∗*^*P* < 0.05; ^*∗∗*^*P* < 0.01.

To determine whether the inhibitory effect of B3GALT5-AS1 overexpression is related to miRNA, we used target prediction software (http://www.microRNA.org/) to predict the sequence of potential B3GALT5-AS1-miRNA interactions, miR-934 was selected as a miRNA with a potential binding site to B3GALT5-AS1 (Figure 5(a)). In order to further explore the association between B3GALT5-AS1 and miR-934 in HCC with luciferase report detection, we constructed B3GALT5-AS1-wt and B3GALT5-AS1-mut clones that have binding sites with miR-934 to pGL3. The experimental result of luciferase activity assay demonstrated the existence of direct interaction between miR-934 and B3GALT4-AS1 (Figure 5(b)). RIP assay revealed that there was specific bind locations of B3GALT4-AS1 on miR-934 (Figure 5(c)). The expression of miR-934 in liver cancer tissues is significantly higher than that in adjacent noncancerous tissues, which is the opposite of B3GALT5-AS1 in HCC (*P* < 0.01, Figure 5(d)). We also examined the expression of miR-934 in five human HCC cell lines (Hep3B, Focus, YY-8103, HCCLM3, and Huh7) and compared it to that in the normal liver cell line L02. The statistical analysis demonstrated that miR-934 expression was higher in HCC cells when compared with L02 cells and the expression of HCCLM3 and Hep3B was relatively highest and lowest, respectively (Figure 5(e)). Furthermore, the results showed that miR-934 expression was significantly reduced after the overexpression of B3GALT5-AS1 in HCCLM3 cells but was upregulated after the knockdown of B3GALT5-AS1 in Hep3B cells (*P* < 0.05, *P* < 0.05; Figures 5(f) and 5(g)). These results suggested that miR-934 was possibly a downstream target of B3GALT5-AS1 and was negatively regulated.

### 3.6. miR-934 Mimics Reverse the Inhibiting Effect of B3GALT5-AS1 ov in HCCLM3

**Figure 6:**
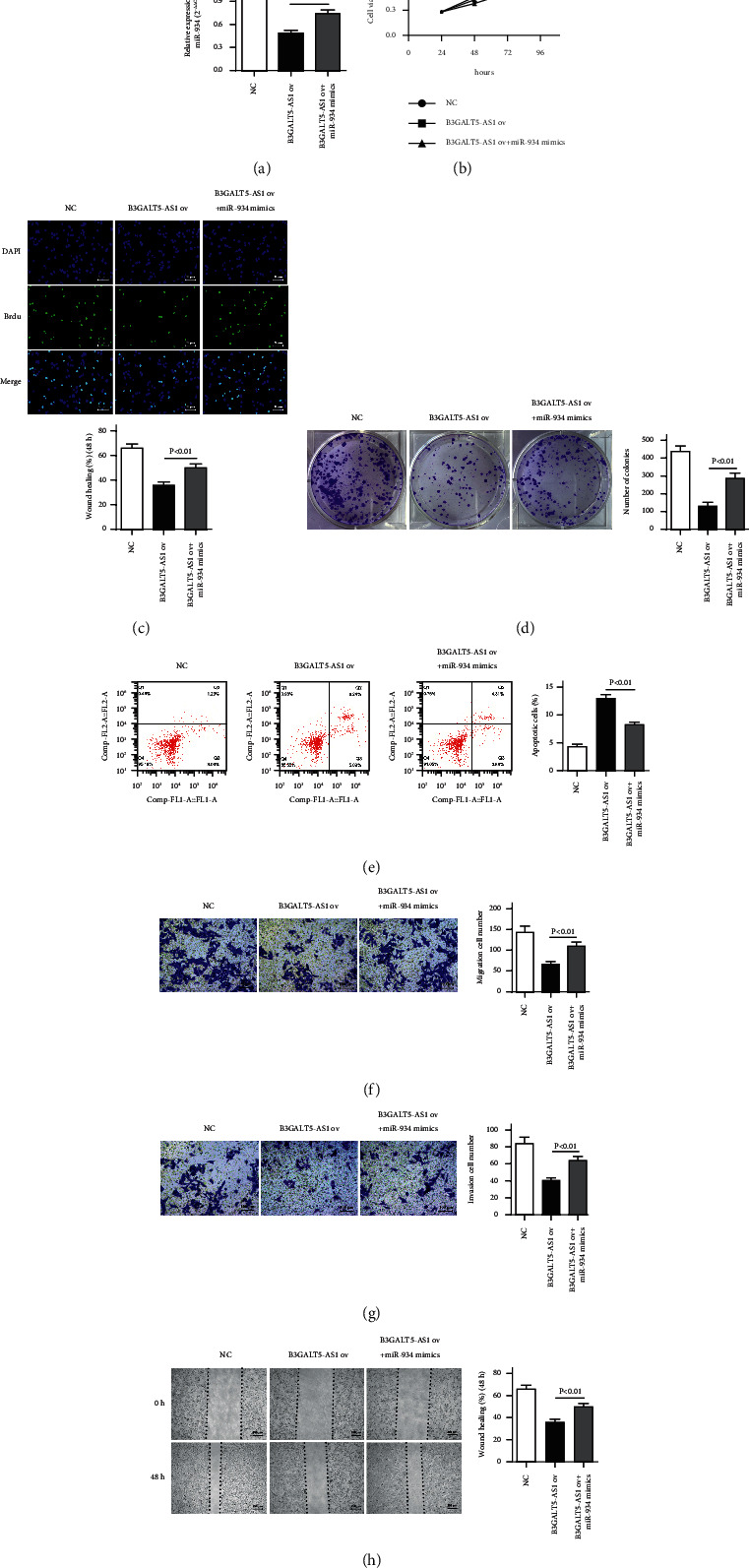
MiR-934 mimics reverse the inhibiting effect of B3GALT5-AS1 ov in HCCLM3. (a) miR-934 expression of NC, B3GALT5-AS1 ov, and B3GALT5-AS1 ov + miR-934 groups. Cell viability detected by CCK-8 assay (b) and BrdU assay (c). Cell colony formation assays (d) and apoptosis assays (e) of NC, B3GALT5-AS1 ov, and B3GALT5-AS1 ov + miR-934 groups. miR-934 had the opposite effect of B3GALT5-AS1 and could reversely regulate the inhibitory effect of B3GALT5-AS1 on cell proliferation. The migration (f) and the invasion (g) ability of HCCLM3 cells. (h) Representative images of wound-healing assays of cells. ^*∗*^*P* < 0.05; ^*∗∗*^*P* < 0.01.

To observe the reversal effect of miR-934 mimics on B3GALT5-AS1 ov in the HCCLM3 cell line, we first detected the expression of miR-934 in three groups (NC, B3GALT5-AS1 ov, and B3GALT5-AS1 ov + miR-934 mimics) after transfection. After adding miR-934 mimics, the expression level of miR-934 was significantly higher than that of the B3GALT5-AS1 ov group (*P* < 0.01; Figure 6(a)). To verify the effects of miR-934 combined with B3GALT5-AS1 on HCCLM3 cell proliferation, migration, and invasion, CKK-8 assay (*P* < 0.05; Figure 6(b)), BrdU assay (*P* < 0.01; Figure 6(c)), cell colony formation assay (*P* < 0.01; Figure 6(d)), flow cytometry (*P* < 0.01; Figure 6(e)), Transwell migration experiment (*P* < 0.01; Figure 6(f)), Transwell invasion experiment (*P* < 0.01; Figure 6(g)), and wound-healing assay (*P* < 0.01; Figure 6(h)) were performed. All the results indicated that miR-934 had the opposite effect of B3GALT5-AS1 and could reversely regulate the inhibitory effect of B3GALT5-AS1 on cell proliferation, migration, and invasion. In summary, the addition of miR-934 significantly weakened the effect of B3GALT5-AS1 ov, indicating that miR-934 mimics could reverse the inhibiting effect of B3GALT5-AS1.

### 3.7. UFM1 Was Identified as a Target of miR-934

**Figure 7:**
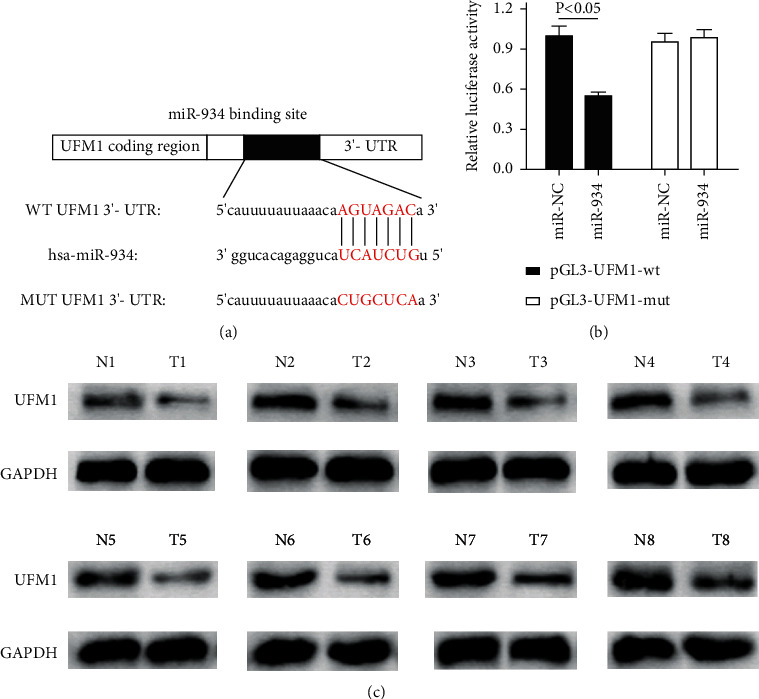
UFM1 was identified as a target of miR-934. (a) Wild-type and mutant-type display of UFM1 3′UTR and miR-934 binding sequence. (b) Luciferase assays of HCC cells cotransfected with the pGL3-UFM1-wt or pGL3-UFM1-mut and miR-NC or miR-934. The assay showed the inhibitory effect of miR-934 on pGL3-UFM1-wt luciferase activity in HCC cells. (c) UFM1 expression in adjacent tissue and tumor tissue measured by western blot analysis. The UFM1 expression in tumor tissues was significantly lower than that in adjacent tissues. ^*∗*^*P* < 0.05.

To study the mechanism of miR-934 interacting with UFM1 *in vitro*, the wild-type and mutant-type sequences of UFM1 3′UTR bound to miR-934 were determined, and we found that there is an overlapping sequence between wt-UFM1 and miR-934 (Figure 7(a)), which suggested the interaction possibility of these two factors. To further explore the correlation by which UFM1 regulated miR-934 expression in HCC, we arranged luciferase activity assays to detect the association between UFM1 and miR-934. As shown in Figure 7(b), pGL3-UFM1-wt plasmid and pGL3-UFM1-mut plasmid were cotransfected with miR-NC or miR-934 into HCC cells. Compared with the pGL3-UFM1-wt and miR-NC cotransfection group, the pGL3-UFM1-wt plasmid and miR-934 mimics cotransfection significantly reduced the luciferase activity of the reporter plasmid, which indirectly elucidated the interaction of UFM1 and miR-934. Those results collectively illustrated that UFM1 is a regulatory target of miR-934. Additionally, the western blot results of 8 pairs of samples showed that the UFM1 expression in tumor tissues was significantly lower than that in adjacent tissues (Figure 7(c)). To sum up, the present results illustrated that UFM1 was a target of miR-934.

### 3.8. UFM1 Downregulation Reverses the Inhibiting Effect of miR-934 Inhibitor in HCCLM3 Cells

**Figure 8:**
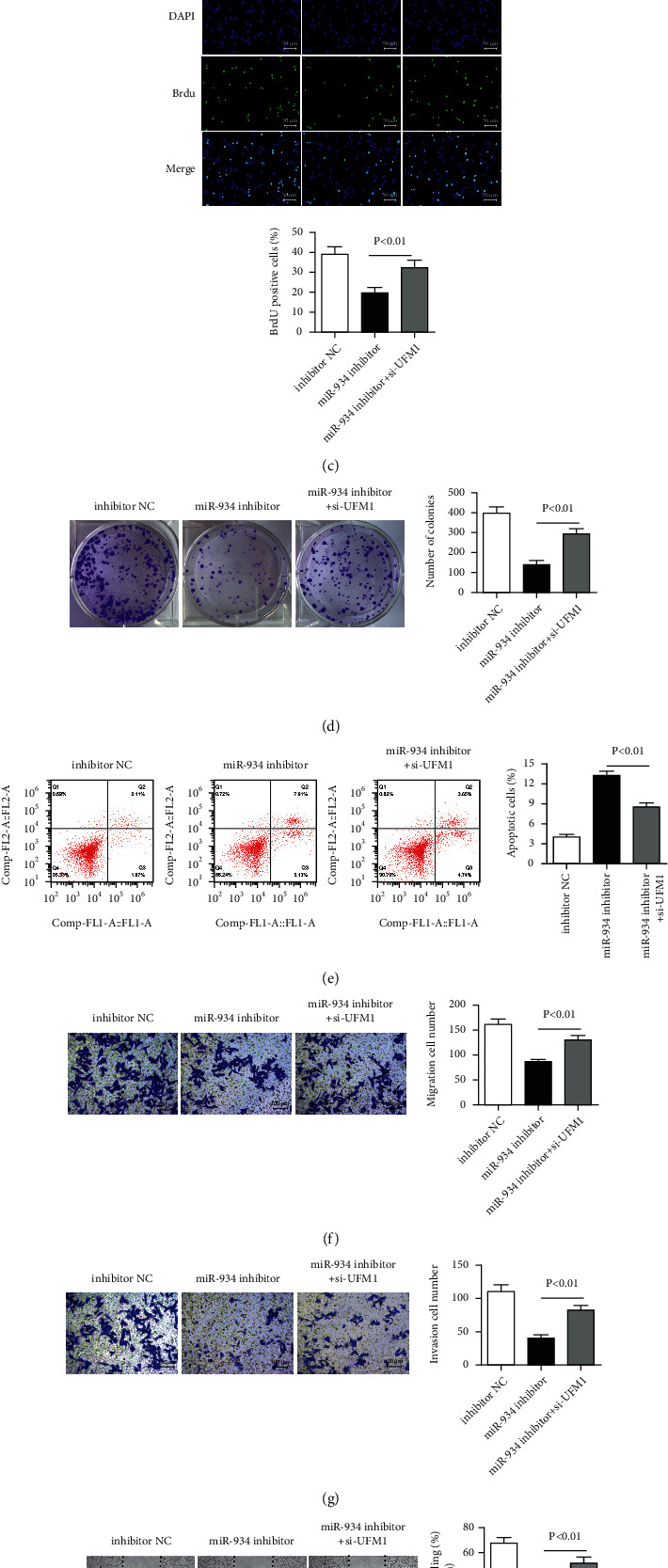
UFM1 downregulation reverses the inhibiting effect of miR-934 inhibitor in HCCLM3 cells. (a) UFM1 mRNA expression of inhibitor NC, miR-934 inhibitor, and miR-934 inhibitor + si-UFM1 groups. Cell viability detected by CCK-8 assay (b) and BrdU assay (c). (d) Cell colony formation assay of each group. (e) Apoptosis assays analyzed by flow cytometric provided that si-UFM1 could reverse the enhanced cell apoptosis ability brought by miR-934 inhibitor. The migration (f) and the invasion (g) ability of HCCLM3 cells. (h) Wound-healing assays exhibited augmented healing ratio in the cotreatment group than in solo miR-934 inhibitor-treated group. ^*∗*^*P* < 0.05; ^*∗∗*^*P* < 0.01.

To investigate the adverse effect of si-UFM1 on the miR-934 inhibitor in the HCCLM3 cell line, we measured the expression of UFM1 in three groups (inhibitor NC, miR-934 inhibitor, and miR-934 inhibitor + si-UFM1) after transfection. The UFM1 mRNA expression level of the miR-934 inhibitor + si-UFM1 group was notably lower than that of only miR-934 inhibitor group (*P* < 0.01; Figure 8(a)). To verify the specific effects of UFM1 in hepatocellular carcinoma (HCC) and its influence toward miR-934, which include the proliferative, invasive capacity alteration with or without UFM1 silencing. CCK-8 assay further indicated that the suppression of miR-934 alleviated cell viability, while the downregulation of UFM1 could reverse this effect (Figure 8(b); ^*∗*^*P* < 0.05), which indirectly suggested that UFM1 was a tumor suppressor in HCC cells. Further verification of this effect of FM1 was conducted using BrdU assay (Figure 8(c); *P* < 0.01) and colony formation assay (Figure 8(d); *P* < 0.01). Cell apoptosis results analyzed by flow cytometric provided that si-UFM1 could reverse the enhanced cell apoptosis ability brought by the miR-934 inhibitor (Figure 8(e)). Next, the invasive capacity of HCC was determined in cells with solo miR-934 inhibitor treatment or cotreatment of miR-934 inhibitor and si-UFM1 through Transwell assay and wound-healing assay. As shown in Figure 8(f), there was decreased cell migration in HCC cells with miR-934 suppression, but increased migration was presented in cells with UFM1 downregulation, which indicated si-UFM1 could reverse the decreased cell migration ability by the miR-934 inhibitor (*P* < 0.01). Similar effects were also observed in cell invasion (Figure 8(g); *P* < 0.01). Moreover, wound-healing assay exhibited augmented healing ratio in the cotreatment group than in the solo miR-934 inhibitor-treated group (Figure 8(h); *P* < 0.01). Conclusively, our experimental results collectively showed that UFM1 partakes in mediating HCC development progress.

### 3.9. B3GALT5-AS1 ov Inhibits the PI3K/AKT Pathway

**Figure 9:**
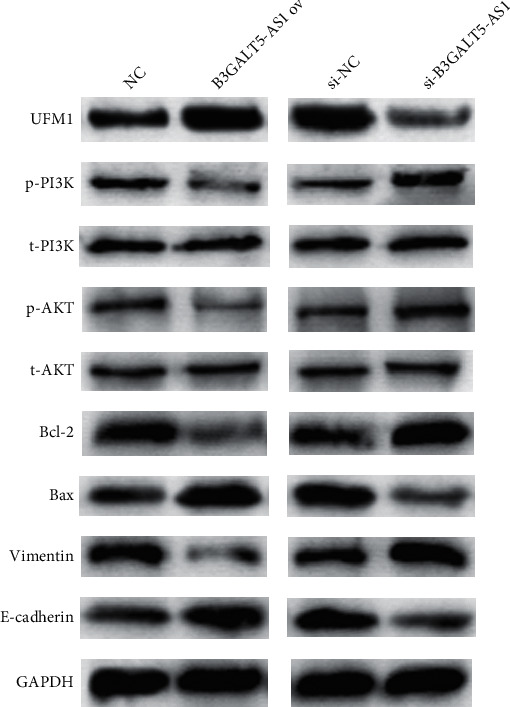
B3GALT5-AS1 inhibits the PI3K/AKT pathway through the UFM1. Western blot analysis of UFM1, p-PI3K, t-PI3K p-AKT, t-AKT, Bcl-2, Bax, Vimentin, and E-cadherin in cells cotransfected with NC or B3GALT5-AS1 ov and si-NC or si-B3GALT5-AS1. B3GALT5-AS1 ov induced by UFM1 resulted in downregulation of p-AKT and p-PI3K and the apoptosis pathway-related proteins Bcl-2 downregulation and Bax upregulation. Meanwhile, EMT pathway-related proteins E-cadherin upregulation and Vimentin downregulation were also observed. B3GALT5-AS1 silencing was the opposite of the above.

In order to illustrate how B3GALT5-AS1 mediates the tumor behavior of HCC, we tested the activity of PI3K/AKT pathway, which is closely related to the proliferation and invasion of HCC cells. Western blot analyses revealed the expression levels of various proteins related to the activity of PI3K/AKT pathway. As shown in Figure 9, in addition to increased UFM1 expression when B3GALT5-AS1 ov in HCC cells, both of the phosphorylated AKT (p-AKT) and the phosphorylated PI3K (p-PI3K) decreased compared with the NC group and further affected the apoptosis pathway-related proteins Bcl-2 downregulation and Bax upregulation. Meanwhile, EMT pathway-related proteins are also affected, including E-cadherin upregulation and Vimentin downregulation. However, after we treated the si-B3GALT5-AS1, we found not only the increased phosphorylation of AKT and phosphorylation of PI3K but also the apoptosis pathway-related proteins Bcl-2 upregulation and Bax downregulation. Meanwhile, we also found the EMT pathway-related proteins E-cadherin downregulation and Vimentin upregulation. In summary, results suggested that B3GALT5-AS1 could suppress the EMT and promote apoptosis of HCC cells through suppressing the PI3K/AKT pathway, as shown in Figure 9.

## 4. Discussion

Liver cancer is one of the deadliest cancers in the world, and HCC accounts for more than 90% of liver cancer cases [21]. Radical resection is currently the preferred treatment for HCC, but most patients have undergone metastases in the liver and circulatory system during surgery. Therefore, the continuous exploration of the molecular mechanisms of cancer occurrence and development and early intervention at the molecular level have become clinically urgent problems.

LncRNAs are a variety of noncoding RNAs containing the largest human genome, with transcripts exceeding 200 nucleotides in length. Although only 1% to 2% of transcription factors have the ability of protein translation, lncRNAs can participate in the body's epigenetic regulation through multiple channels such as chromatin remodeling, transcription regulation, and posttranscription [22, 23]. HCC is a primary liver cancer with high mortality, so it is very important to find suitable therapeutic targets. Up to now, the expressions of lncRNA UCID [24], Tim3 [25], PDZD7 [26], DILC [27], FTX [28], ELF209 [29], HC [30], and HULC [31] indicate influential prognoses of HCC patients. B3GALT5-AS1 is the newly identified lncRNA, and the downregulation of B3GALT5-AS1 is often observed in human cancers. Accumulating data have indicated that the expression levels of lncRNA B3GALT5-AS1 are also reduced in colon cancer. Wang et al. found that the decreased expression of B3GALT5-AS1 in colon cancer tissues caused the metastasis and invasion of colon cancer cells, and the decreased expression of B3GALT5-AS1 is related to the poor prognosis of colon cancer patients [11]. Herein, lncRNA B3GALT5-AS1 expression levels were diminished in HCC compared with normal tissues and displayed a negative correlation, indicating that lncRNA B3GALT5-AS1 might be a negative factor in HCC, and the knockdown of B3GALT5-AS1 favors HCC cell proliferation, migration, and invasion, and the downregulation of lncRNA B3GALT5-AS1 can be used to predict survival in HCC.

More and more evidence shows that many dysregulated miRNAs exhibit carcinogenic or tumor suppressor effects on the occurrence and development of HCC and are potential diagnostic markers and new therapeutic targets for HCC [32, 33]. According to reports, miR-934 has a promoting effect on the occurrence and development of a variety of cancers, but its role in liver cancer has not been found so far [34]. Jin et al. reported the mechanism of miR-934 in pancreatic cancer. Their results showed that miR-934 upregulation is related to the prognosis of pancreatic cancer patients and that the increase in cellular malignant characteristics caused by miR-934 overexpression can be reversed by inhibiting the expression of PROX1 [34]. Hu et al. reported that miR-934 was significantly increased in ovarian cancer cell lines and downregulated miR-934 remarkably inhibited cell progression. In addition, they identified BRMS1L as a target gene of miR-934 [35]. MiR-934 is a promising tumor treatment target, but its interaction mechanism with lncRNA is not clear, and our research has solved this confusion. UFM1 is a member of the ubiquitin-like modifier (UBL) family and exists in almost all eukaryotic cells. Komatsu et al. first discovered UFM1 in HEK293 cells in 2004 [36]. UFM1 may be related to lncRNA and play an important role in the progression of tumors. Therefore, the mechanism of UFM1 and miR-934 in B3GALT5-AS1 in HCC has also been explored together in our study.

In conclusion, our findings demonstrated that lncRNA B3GALT5-AS1 restrained HCC cell progression through regulating the miR-934/UFM1 axis and represent a potential therapeutic target. B3GALT5-AS1 ov can inhibit tumor progression *in vitro* and *in vivo*, and silencing B3GALT5-AS1 enhanced the expression of miR-934, while B3GALT5-AS1 ov exerted an opposite effect. In terms of the association of miR-934 and UFM1, we found that lncRNA B3GALT5-AS1 negatively regulated miR-934 to inhibit HCC cell growth and UFM1 showed the opposite effect of miR-934. Moreover, we revealed that B3GALT5-AS1 ov inhibited the PI3K/AKT pathway to suppress HCC. Our findings proved that B3GALT5-AS1 might be a potential therapeutic target for HCC treatment in the future.

## Figures and Tables

**Table 1:** Primers used in this study.

Name	Sequences
B3GALT5-AS1 forward	5′-GATCCACGTCCAGGCTCACT-3′
B3GALT5-AS1 reverse	5′-GTGCTGGCTGTCAGGATGAG-3′
miR-934 forward	5′-AGGGTGTCTACTACTGGAGA-3′
miR-934 reverse	5′-GTTGTGGTTGGTTGGTTTGT-3′
UFM1 forward	5′-TCGGAAGTGCTGATGAGTT-3′
UFM1 reverse	5′-CCTCCTTAATAGAAGCCTGGT-3′
U6 forward	5′-CTCGCTTCGGCAGCACA‐3′
U6 reverse	5′-GGATGGTGATGGTTTGGTAG‐3′
GAPDH forward	5′‐TCCTCTGACTTCAACAGCGACAC‐3′
GAPDH reverse	5′-CACCCTGTTGCTGTAGCCAAATTC‐3′

**Table 2:** Correlation of B3GALT5-AS1 expression with clinicopathologic characteristics in HCC patients.

Characteristic	Case	B3GALT5-AS1 expression		*P* value
		Low	High	
All case	56	28	28	
Age (years)				0.584
<60	22	10	12	
≥60	34	18	16	
Gender				0.788
Female	25	12	13	
Male	31	16	15	
Tumor size (cm)				0.022^*∗*^
<5 cm	38	23	15	
≥5 cm	18	5	13	
Liver cirrhosis				0.313
No	11	4	7	
Yes	45	24	21	
HBsAg status				0.515
Negative	12	5	7	
Positive	44	23	21	
AFP (ng/ml)				0.108
≤20	26	16	10	
>20	30	12	18	
Tumor multiplicity				0.383
Single	39	21	18	
Multiple	17	7	10	
Edmondson grade				0.42
I–II	31	17	14	
III–IV	25	11	14	
TNM stage				0.003^*∗*^
I–II	33	22	11	
III–IV	23	6	17	

## Data Availability

All data generated or analyzed during this study are included within this article.
